# The Role of Hybrid Varieties in Enhancing Crop Productivity and Sustainability in Nepalese Agriculture

**DOI:** 10.1155/sci5/8275428

**Published:** 2025-07-21

**Authors:** Dipak Raj Bist, Adhiraj Kunwar, Pawan Chapagaee, Lokendra Khatri, Bibek Bhatt, Ashmita Mandal

**Affiliations:** Gokuleshwor Agriculture and Animal Science College, Tribhuvan University, Baitadi, Nepal

**Keywords:** agricultural sustainability, crop productivity, environmental resilience, food security

## Abstract

Nepal is an agrarian-based country with most of the population engaged in the agriculture sector for their livelihood. Nepal, with its numerous agroecological zones, is moving from subsistence to commercial agriculture to reduce poverty, provide food security, and improve economic growth. Crop breeding programs were initiated in 1951 A.D. in Nepal to focus on the development of varieties for cereal crops, but most of the farmers are still based on their low-yielding traditional varieties. Hybrid varieties, developed by the cross-pollination between different plant varieties, play a vital part in this change by demonstrating heterosis, resulting in increased yields, resilience to biotic and abiotic challenges, and consistency in agronomic features. Hybrid maize and rice exhibit considerable potential to increase productivity, enhance smallholder farmers' earnings, and strengthen food security via efficient farming methods and mechanization. Despite these advantages, Nepal's dependency on imported hybrid seeds highlights issues such as expensive fertilizer and pesticide input costs, limited local production capacity, and concerns about genetic erosion. Nepal mostly imports hybrid seeds, and due to the rising demand, the country allocates substantial financial resources for their importation. In Nepal, the low quality of hybrid seed imported by different seed agencies and companies causes yield loss, more expenditure by farmers for purchasing seed, and a higher incidence of insect, pest, and disease. The Nepalese government should place emphasis on conducting research on hybrid seed development for cereal crops as well as vegetable seeds in collaboration with the private sector.

## 1. Introduction

Nepal, an agricultural country with diverse ecological regions, is home to nearly 30 million people, with 57.3% actively engaged in agriculture, including 50.6% males and 64.8% females. Agriculture is the backbone of Nepal's economy, providing livelihoods and contributing 24.1% of total GDP [1]. The Nepalese government evaluates the agricultural industry annually and assigns significance to agricultural objectives, laws, and budgets for each year. Nevertheless, the agricultural sector contributing to eliminating poverty is also poor. In developing countries like Nepal, people engaged in the agricultural sector are comparatively poorer than those engaged in other sectors [2]. Nepal has the lowest crop productivity among South Asian countries, and its agriculture is focused mainly on maintaining itself. However, the government of Nepal promulgated a shift from subsistence to commercial agriculture in the ADS strategy (2015–2025). It will help Nepal eliminate poverty, improve food security, provide employment opportunities to the country's youth, and help to stop youth migration for job opportunities. As Nepal's government prepares to commercialize agriculture, hybrid varieties will be key factors in achieving this milestone [3]. Hybrid crops are produced by crossing two distinct plant kinds, passing on enhanced characteristics from both parents [4]. This technique produces F1 hybrid seeds by fertilizing the female parent with pollen from the male parent. Compared to nonhybrids, hybrids are more robust, productive, and resilient to biotic and abiotic stressors because of their heterosis [5, 6]. Although hybrids can occur in both self-pollinated and cross-pollinated species, cross-pollinated plants are more likely to produce them because of increased heterosis expression [7, 8]. Despite being heterozygous, hybrid genotypes result in populations with consistent features and genetic uniformity.

Hybrid maize and rice have the potential to greatly increase the incomes of smallholder farmers and enhance the food security of households, especially in developing nations [9]. Hybrid crops have demonstrated greater resilience to environmental challenges such as drought, resulting in more efficient use of the growing season and the ability to have multiple cropping cycles within a year [10]. With increasing productivity, hybrid varieties have a significant impact on agriculture systems. The uniformity of hybrid crops aids in mechanization and improves the consistency of agricultural products, making them more appealing to the commercial market. However, the increased use of hybrids also causes crop genetic diversity to be eroded [11]. The agricultural systems of Nepal have been greatly influenced by hybrid varieties due to their increased production. Mechanized harvesting and large-scale production are supported by hybrid maize varieties such as *Rampur Hybrid-2*, which have demonstrated yield gains of up to 30% when compared to open-pollinated varieties (OPVs) [12]. The use of modern technology has been made easier by the homogeneity of hybrid crops, especially in the Terai region's commercial vegetable farming [13]. Concerns over genetic erosion have been raised, though, as the introduction of hybrids has aided in the decrease of traditional landraces, including local maize types and *Jhinuwa rice* [14, 15].

The aim of this review is to examine how hybrid crop types affect Nepalese agriculture, specifically yields, farm incomes, food security, natural diversity, and environmental sustainability. This narrative review will emphasize the advantages and difficulties of hybrid adoption by combining knowledge from previous research, reports, and expert perspectives. It will also provide a critical analysis of the long-term effects of hybrid farming, assisting farmers and decision-makers in making wise choices for Nepal's sustainable agricultural growth.

## 2. Adoption of Hybrid Seeds Among Farming Families

**Figure 1:**
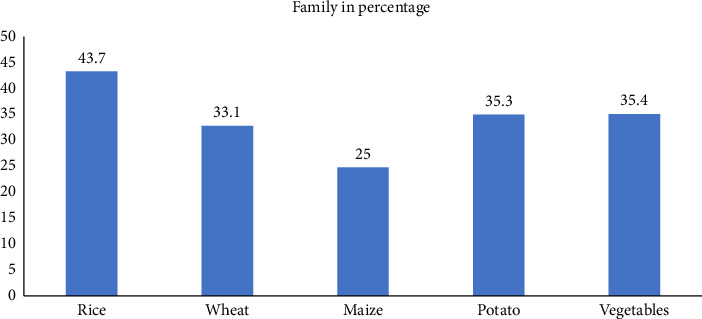
Adoption of hybrid seeds among farming families.

The percentage of families using hybrid seeds for various crops is depicted in the bar chart. With 43.7% of families using hybrid seed varieties, rice has the greatest acceptance rate. With 35.4% and 35.3%, respectively, vegetables and potatoes come next, suggesting a comparatively large usage of hybrids in vegetable crops. Of the crops surveyed, maize has the lowest hybrid seed utilization, with only 25% of families choosing hybrid varieties, while wheat has a slightly lower adoption rate of 33.1%. This graph shows how different crop species have adopted hybrid seeds at different rates, which may be due to a variety of reasons such as farmer preferences, input costs, yield potential, and seed availability (Figure 1) [16].

**Figure 2:**
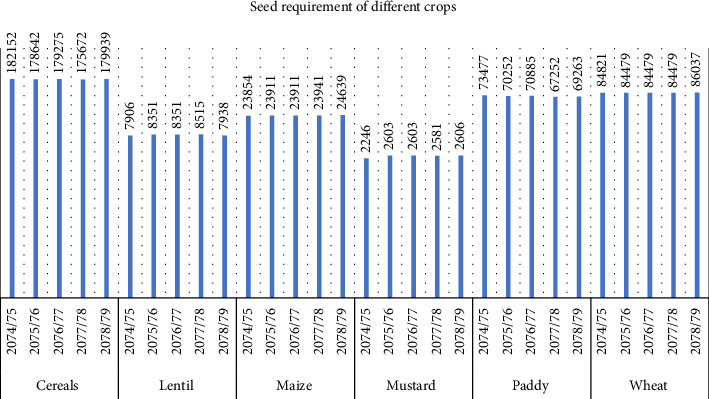
Seed requirement of different crops in Nepal in metric tons.

Figure 2 provides the estimation of the seed requirement of different crops in Nepal. According to the statistical data on Nepalese agriculture, the requirement of cereal crop from FY 2074/079 is decreased to a small degree, but the requirement is highest compared to other crops, which is then followed by wheat. However, the paddy requirement is decreasing, which was 73,000 mt. in FY 2074 but decreased to 69,000 mt. in FY 2079. In the case of maize, the data suggest that there is a relatively equivalent requirement of seeds. Lentil requirement is increased in FY (2075/076), (2076/077), and (2077/078), but in year 2078/079, the requirement is decreased slightly. Mustard has the lowest seed requirement among all other crops [17]. Nepal's agricultural policy should prioritize hybrid seed research, development, and distribution to enhance productivity, ensure food security, and align with changing farmer preferences. The decline in paddy seed demand suggests a shift in cropping patterns, necessitating drought-resistant, high-yield hybrid rice varieties. The stability in maize seed demand indicates continued importance in Nepalese agriculture, while the increasing demand for lentil seeds suggests growing interest in pulses. Interventions like subsidies and market incentives are needed to reduce dependency on oil imports [18].

**Figure 3:**
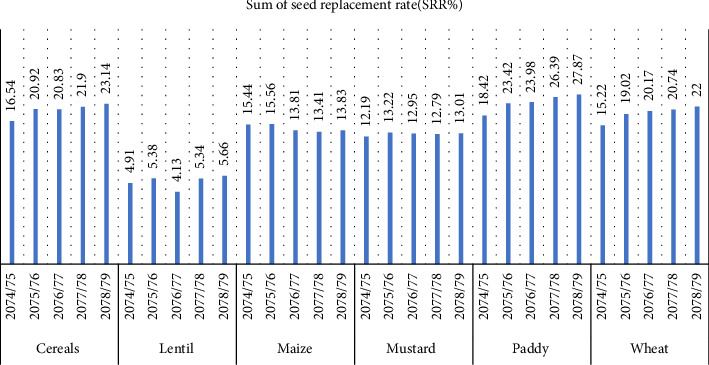
Seed replacement rate of households in Nepal.

Significant advances in the adoption of better seed types and their impact on agricultural output are shown by the variations in Nepal's seed replacement rate (SRR) for different crops between 2074 and 2079. The greatest SRR is constantly displayed by paddy, which rose from 18% in 2074 to 28% in 2079. Because rice is a main crop in Nepal and there is a considerable demand for higher quality seed to address food security demands, this trend can be linked to the government's aggressive promotion of high-yielding varieties and subsidies for enhanced seeds. In order to increase production, programs like the National Seed Vision have also been instrumental in promoting the substitution of hybrid or enhanced rice varieties for conventional ones [17]. The SRR for cereals has been increasing steadily, from 16% in 2074 to 23% in 2079, largely due to growing awareness of modern seed varieties and food security efforts. Wheat has shown a consistent upward trend, increasing from 15% in 2074/075 to 22% in 2078/079. This is attributed to the introduction of hybrid varieties and improved crop management practices. However, maize adoption has declined, falling from 15.44% in 2074/075 to 13.84% in 2078/079. Mustard adoption has been relatively stable, fluctuating between 12% and 13% over the years, possibly due to lower demand, limited research, or insufficient extension services. Lentil adoption has shown minimal change, possibly due to cultural practices (Figure 3). The distribution of SRR in crops is affecting agricultural productivity and sustainability. High SRR in paddy and wheat indicates increased adoption of improved seed varieties, but reliance on external inputs may limit sustainability. Hybrid varieties can lead to genetic erosion and reduced agrobiodiversity. Declining SRR in maize and lentils raises concerns about long-term productivity. A balanced approach involving hybrid and traditional varieties is needed for long-term agricultural productivity [19].

According to the statistical data on Nepalese agriculture 2081, Figure 4 illustrates the total seed supply of different crops. In 2074/075, the total seed supply of cereal crops is on an increasing trend, with 30,000 mt. in FY 2074/075, 36,000 mt. in FY 2075/076, 37,000 mt. in FY 2076/077, 38,500 mt. in FY 2077/078, and 41,000 mt. in FY 2078/079, while in paddy, the supply is 13,000 mt., 16,500 mt., 17,000 mt., 17,500 mt., and 19,500 mt. from 2074 to 2079, respectively, which is also in an increasing trend. However, wheat had a jump in supply of seed of 16,000 mt. in 2075/076 from 13,000 mt. in 2074/075, where the trend went on significantly increasing up to 19,000 mt. in FY 2078/079. Maize seed supply is slightly constant, with an average seed supply of 3500 mt. from 2074-079. Mustard has a low supply of seed: 274 mt in 2074/075, 344 mt in 2075/076, 337 mt in 2077/078, and 339 mt in 2078/079. And finally, lentil supply decreased in FY 2076/077 to 345 mt. from 449 mt. in FY 2075/076, and then, the trend went on increasing, which was 455 mt. in FY 2077/078 and 449 mt. in FY 2078/079 [17]. Government regulations, consumer demand, and studies on hybrid cultivars are some of the variables that impact these patterns. While the stable supply of maize seeds indicates low hybrid adoption, the increasing availability of hybrid seeds for wheat and rice indicates rising adoption of hybrid types due to improved yields and climatic resistance. More research on hybrid variants for mustard and lentils is necessary, as evidenced by the low and erratic seed availability. These patterns indicate that the use of hybrid seeds for important crops is increasing, but in order to increase supply and farmer involvement, noncereal crops require greater funding [20].

## 3. History of Hybrid

Modern agriculture has been revolutionized by the creation of hybrid seeds, especially once farming became more industrialized in the 20th century. In the 1920s, F1 hybrid maize was commercialized in the United States, marking one of the first advances in hybrid seed technology. Crop yields increased significantly as a result, which aided in the development of a global seed business. Particularly during the Green Revolution of the mid-20th century, when high-yielding hybrid crops were widely used, hybrid wheat and rice cultivars substantially increased agricultural production [21]. Research on hybrid rice started in China in 1964, and by 1967, the first commercial hybrid rice was produced [22]. Commercial cytoplasmic male sterile (CMS) hybrid wheat was introduced in the United States in 1974, and in the 1980s, hybrid wheat innovations also appeared in Australia and Europe [23]. European nations have lately redoubled their attempts to create hybrid wheat types suited for commercial success, despite early disappointments [24]. In Nepal, due to changes in its neighbors, especially China and India, Nepal started experimenting with hybrid seed technologies later. In 1987, the National Maize Research Program (NMRP) tested nine hybrid maize types purchased from foreign seed firms with headquarters in India. In an effort to increase food security and economic resilience, Nepal has now created and published its own hybrid maize varieties, such as Rampur Hybrid-10, Rampur Hybrid-12, Rampur Hybrid-14, and Rampur Hybrid-16 [20, 25]. Early in the 1970s, the Agriculture Botany Division (ABD) in Khumaltar started doing research on hybrid wheat in Nepal. Later, the National Wheat Research Program (NWRP) in Bhairahawa took over [26]. Although adoption rates are still lower because to issues with seed multiplication and farmer acceptability, Nepal has caught up to China in the production of hybrid seeds for the rice industry. Hybrid seed adoption in Nepal faces several challenges, including economic dependency, cost barriers, input-intensive farming, loss of traditional landraces, genetic erosion, and limited research and development capacity. Hybrid seeds require constant purchase, increasing financial dependence on seed suppliers, and requiring higher doses of fertilizers, pesticides, and irrigation [27]. This shift has increased vulnerability to climate change and emerging pest pressures. Nepal's reliance on government research institutions and international collaborations also reduces seed sovereignty and dependency on foreign suppliers. Nepal has implemented policies to balance hybrid adoption with sustainability, including the National Seed Vision 2013–2025, subsidies for hybrid seeds, and agrobiodiversity conservation programs. However, ensuring equitable access remains a challenge. A balanced approach integrating hybrid seed technology with traditional variety conservation, public–private partnerships, extension services, and climate-resilient hybrid varieties could enhance the long-term sustainability of hybrid adoption in Nepal [28].

## 4. Present Status of Hybrid Seed Production in Nepal

Since 2018, the Nepal government has not been actively involved in developing hybrid varieties, so private seed manufacturing companies conduct varietal testing and production. The government must approve a licensing scheme for assigning hybrid maize varieties to the private sector, as many seed firms lack the necessary resources for hybrid creation [29]. Subash Raj Upadhyaya, chairperson of Lumbini Seed Company, began planting hybrid maize in 2018 after receiving training and technical support from USAID's Feed the Future Nepal Seed and Fertilizer activity. The company aims to produce 50 ha by 2023 and 200 metric tons by 2022 [30]. Nepal, once a seed-exporting country, now imports over 90% of vegetable seeds, 30% of maize seeds, and 15% of rice seeds. In 2018/2019, the country imported 424,333 kg of vegetable seeds worth Rs 553.08 million and 4.22 million kg of maize seeds worth Rs 393.16 million [31]. There is a yearly increase in demand for improved and hybrid vegetable seeds. The commercial vegetable-producing pockets cover more than 60% of the land, and the use of hybrid seeds is growing because of their superior yielding capacity over open-pollinated (OP) types [32]. The use of hybrid varieties of major vegetable crops like cabbage, tomato, cauliflower, cucurbits, onion, and carrot is increasing every year in Nepal. About 73% of the vegetable production area is estimated to be covered by hybrid varieties in Nepal. A huge volume of hybrid seeds of vegetable crops is imported from India, China, Thailand, Japan, Korea, and the Netherlands [33].

## 5. Advantages of Hybrid Seed

The main advantages of hybrid seed are that the income generated from corn hybrid seed production is greater than that of corn seed production from farmer-saving seed, and the B:C ratio shows corn hybrid seed production is profitable. This is the strongest factor in attracting farmers because farmers are profit-oriented [32]. The main advantage of hybrid rice is a perceived higher yield compared to inbred varieties. The productivity of hybrid rice varieties is higher than that of traditional varieties, despite the higher yield agrochemically, and other inputs like fertilizer, irrigation, and pesticides are also generally required in higher amounts for hybrid rice than for conventional rice varieties [34, 35]. Hybrid rice genotypes have not only high-yield potential over inbred varieties, but also distinctive features, such as being highly responsive to fertilizer and showing more adaptability in the new environment. Hybrid seeds have many attractive features, including being more stable in adverse climatic conditions such as drought, stress, and wind, as well as being suitable for mechanized harvesting. Hybrid varieties are more suitable and easier to apply to machines for harvesting, fertilizer application, and pesticide application [36]. Hybrid maize in Nepal has shown 25%–30% increase in yield compared to traditional varieties, with higher benefit–cost ratios. Farmers in Kavrepalanchok and Sindhupalchok districts found fertilizer responsiveness and wind stress advantages. Despite increased input costs, hybrid maize offers profitability [37]. Hybrid rice varieties in Nepal's Terai region have shown significant yield gains, with 20%–25% higher yields than inbred varieties. They are highly responsive to fertilizer and perform better under drought stress. However, increased pesticide and fertilizer use raises production costs, impacting profitability. Despite these challenges, hybrid rice yields are justified for many farmers [38]. Hybrid tomato varieties in Chitwan, like Srijana, are popular due to their resistance to pests and diseases, high yield, and uniform size. Despite higher seed costs, they yield 40%–50% more per hectare, reduce labor costs, and are highly valued in urban markets for their long shelf life and appearance [39]. The benefits of hybrid seeds in various Nepali agricultural situations are examined in further detail in these case studies. They acknowledge the difficulties associated with input costs and management while highlighting not just the yield increases but also the reaction to inputs, flexibility under stress, and market attractiveness.

## 6. Disadvantages of Hybrid Seed

The one major drawback of hybrid seed is that seed from the first generation of hybrid plants does not consistently yield true copies; hybrid seed cannot be stored; and fresh seed has to be bought for every planting season [40]. All of the hybrid seeds planted by the farmer will yield similar plants, whereas the seeds of the following generation from those hybrids will not consistently have the desired qualities. Controlled hybrids give relatively homogeneous traits because they are developed by crossing two inbred strains [5]. Hybrid seeds are often more concerning for farmers because they require more cost than traditional seeds. Hybrid varieties require more agrochemical and other inputs, like more fertilizer and pesticides. Also, the main thing about hybrid seed is that the price per kg of hybrid seed is relatively higher than that of traditional seed or farmers' saved seeds of maize crops [41]. Hybrid maize adoption in Nepal's mid-hill regions has several disadvantages, including higher yields but additional costs, reliance on chemical fertilizers and pesticides, and financial burdens for farmers. Despite improved yields, fluctuating market prices and nonreproducibility of hybrid seed further exacerbate the situation, as farmers must purchase fresh seed every season, making it a continuous financial commitment [42]. Despite its higher yield potential, hybrid rice in the Terai region faces challenges for farmers. Despite outperforming traditional varieties, hybrid rice requires reinvestment in seeds, high seed prices, increased reliance on agrochemicals, and a lack of seed-saving capability. This financial strain leads to greater reliance on external suppliers and a strain on farmers' resources. The inability to reproduce is the main drawback of hybrid seeds. Because hybrid seeds do not yield true-to-type progeny, farmers must purchase new hybrid seed each season, unlike conventional types where they can conserve seeds for later planting seasons. Many smallholder farmers find it difficult to pay for the ongoing expenses that result from this. In order to sustain good yields, hybrid cultivars frequently need additional agrochemicals (fertilizers, insecticides, and herbicides), which drives up production costs even further. Farmers, especially those with limited means, experience financial hardship as a result of these input costs and rising seed prices [20].

## 7. Perception of Farmers Toward Adoption of Hybrids

Only increasing in yield is not the sole factor for acceptance of hybrid varieties by the farmers; their grain quality traits make more concern for the acceptance of consumers because nowadays consumers are more aware of quality than quantity. For farmers, quality equates to profitability. If quality is not maintained, then farmers fetch a low price in the market. For the development of hybrid seeds in rice breeding programs, there should be an emphasis on improving an appreciable amount of grain quality [43]. Small-scale farmers can save money by using locally available high-yielding varieties with small profit margins by reducing the cost of chemicals because the cost of production for hybrid seed is greater than that of local varieties. This is due to fertilizer and pesticide requirements for hybrids being high, and the price of chemical input is increasing. Farmers have not operated costs, so they required credit for next-season planting. If they reduce the cost of cultivation, then directly reduce the cost. For this reason, farmers are interested in local high-yielding varieties [44]. The perception of farmers for the selection of hybrid seed is due to higher production, attractive fruits, quality seed, and more profit, whereas the main reason for choosing OPVs is due to easy availability and slightly less incidence of insect pests than hybrid seed, and the preference given by the consumer for vegetable seed is taken for research by Timsina and Shivakoti [45]. Nepali farmers' preference for hybrid seeds may be changing due to a 2011 outcry over Monsanto's subsidized distribution plan in three districts. This has led to the company losing ground to competitors. Meanwhile, more Nepali farmers and consumers are realizing the importance of growing organic crops but not abandoning hybrids entirely. Locals often prefer tomatoes that taste and smell like their original counterparts, unlike hybrids that may lose flavor due to size, color, or other traits. This affects not only farmers and consumers but also concern for everyone. Native varieties of tomatoes continued to be popular in urban markets due to their superior flavor and scent, but hybrid tomatoes dominated commercial fields due to their appealing size and shelf life. The study came to the conclusion that in order for hybrid adoption to be effective, productivity and customer-preferred quality attributes had to be balanced [46]. Traditional varieties may be preferred by customers since hybrid crops with greater physical attributes (uniform size, vivid color) may not necessarily have the desirable flavor. For instance, native tomato varieties are frequently preferred by Nepali customers owing to their fuller flavor, whereas other hybrid tomatoes have been condemned for having poor taste because of their breeding emphasis on size and shelf life [47]. The demand for organic crops among consumers has changed as a result of increased knowledge of environmental and health issues. Despite the increased yields of hybrid types, farmers are increasingly choosing to cultivate OPVs that require fewer chemical inputs. The demand for locally adapted OP vegetables has expanded as a result of consumers' desire for naturally cultivated vegetables [48]. The adoption of hybrid seeds was greatly impacted by the 2011 controversy over Monsanto's sponsored hybrid seed distribution scheme in three regions of Nepal. Monsanto's market share decreased as a result of the reaction against genetically modified (GM) seeds, and local farmers were more dubious of foreign hybrid types. Additionally, this event strengthened farmer and consumer interest in OP and indigenous cultivars [49]. Hybrid rice types produced yields that were 15%–25% greater than those of traditional varieties, but their uptake was still restricted because of subpar grain quality characteristics including flavor and cooking texture. Due to their inability to sell hybrid rice at high rates, many farmers turned back to native kinds that were more valuable on the market [50]. Due to its great production potential, hybrid maize was first embraced by farmers in Nepal's mid-hill areas. Customers were resistant to hybrid maize since its flour quality was inferior to that of conventional types. Because of this, the adoption of hybrid maize stayed confined in regions where the demand for animal feed exceeded worries about human consumption [42].

## 8. Marketing Channel for Hybrid Seed in Nepal

In Nepal, hybrid seeds are not developed in an appreciable amount. Mostly, Nepal depends on other countries for hybrid seed. Very few of the works are done in the sector of hybrid variety development. The requirement for hybrid seed is fulfilled in Nepal by imports from other countries. Cereal crop hybrid seed (maize and rice) usually enters through Nepal–India's open border channel. Hybrid vegetable seeds are imported from different countries: Thailand, China, Korea, Japan, and India. Presently, nearly 30 foreign companies are involved in supplying the seeds of crops like rice, maize, and vegetables to Nepal. The growing demand for hybrid seeds is directly related to this seed marketing channel structure. From 2010 to 2025, the area under hybrid maize cultivation is projected to grow from 85,000 to 250,000 ha, with seed requirements increasing from 1275 to 3750 metric tons, similar to rice and vegetables shown in Table 1. This increment in seed requirement and area under cultivation of hybrid seed highlights the expanding market potential and importance of an efficient marketing and distribution system.

The primary focus of seed business activities is on the delivery and sale of next generation seeds, which are generated by various individuals and organizations. These seeds are mostly offered via seed dealers and merchants. The Radha-4, Sabitri, and US 312 (rice hybrid) are the most popular types with the biggest seed sales. Because of their higher market margins, seed dealers supplying hybrids enjoy greater rewards. The principal restrictions faced by seed actors are poor profit margins in seed sales of domestic varieties as opposed to exotic hybrids [51].

## 9. Impact of Hybrid Varieties on Local Agrobiodiversity

Due to the fast growth of contemporary agricultural systems and the extensive use of commercial hybrid and GM crops, almost 75% of the world's genetic diversity has been lost during the past 20 years [52]. Hybrids are developed through cross-pollination by carefully selecting parents, which reduces genetic diversity. Hybrid varieties are developed under the control of plant breeders. Traditional varieties are different from hybrid seeds and GM organisms in some respects. Traditional varieties are a tool for conserving the agrobiodiversity. They are derived from wild plants; they are domesticated and planted locally based on local indigenous knowledge [53]. The global agricultural market favors high-yielding, commercially viable crops, leading to the displacement of traditional landraces. Economic forces include profit maximization, consumer preferences, and export-oriented agriculture, with hybrid rice varieties replacing indigenous landraces in developing countries like Nepal. Multinational corporations dominate the global seed market, reducing on-farm genetic diversity and displacing local varieties. Farmers purchase hybrid and GM seeds, leading to the disappearance of indigenous cotton varieties in countries like India [54]. Government support for modern agriculture encourages hybrid and GM crops over traditional ones through input subsidies, research priorities, and extension services bias. For example, China's hybrid rice program has led to the loss of over 60% of traditional varieties [55]. Land fragmentation, urban migration, and climate change adaptation are causing farmers to prioritize hybrid crops, loss of traditional knowledge, and reduced genetic diversity in local fields [56]. Policy factors such as intellectual property rights, seed patents, and UPOV agreements contribute to the loss of genetic diversity in agriculture. These laws limit farmers' ability to save and exchange seeds, reducing genetic diversity. Policies promoting monoculture and large-scale farming also contribute to soil degradation and pest vulnerability. Agricultural trade policies prioritize the mass production of staple crops, leading to genetic erosion in traditional crops. Lack of support for agrobiodiversity conservation, limited research on indigenous crops, and lack of incentives for traditional farming further contribute to this issue [57]. The main reason for the loss of genetic diversity in modern agriculture is the shift from traditional agriculture to modern agriculture, commercial agriculture, and the adoption of new varieties. There are two main reasons for the erosion of genetic diversity: The first is the replacement of landraces by modern high-yielding cultivars, and the second is selective artificial breeding because breeders are only interested in desirable traits [58]. The use of high-yielding hybrid varieties, synthetic fertilizer, and pesticides negatively impacts the environment due to the heavy use of fossil fuels and natural resources in modern agriculture. The deterioration of natural habitats causes soil and water degradation as well as a variety of residual effects on the surrounding environment [59].

## 10. Positive Impact of Hybrid in Crop Production

Adapting high-yielding hybrid varieties to replace older ones is critical for the country's economic and population growth. Genetics and plant breeding are important techniques for increasing productivity in agriculture, nutritional quality, and food security through development hybrid varieties. In Bangladesh, the adaptation of hybrid rice varieties increased rice yield and technical efficiency by 12.03% and 6.24%, respectively [60]. In the Philippines, hybrid rice produces a higher yield than inbred rice, facing a higher price in the market. This led to a higher gross income from hybrid rice farming, as reported by [61]. Reports show that hybrid rice varieties outperform conventional rice varieties in both agronomic and economic performance, suggesting greater potential for hybrid rice varieties [62]. Delayed planting shortens hybrids' time to silking, lowering the amount of time spent in vegetative growth. This permits hybrids to adjust for shorter growing seasons due to delayed planting. The decreased time to silking balances the increased time to physiological maturity [63]. A corn hybrid with a short growth period can be harvested within a span of 90–100 days after sowing (DAS). This enables many crop cycles in a year and enhances crop productivity [64]. Studies conducted in Nepal reveal a similar pattern. In the Terai and mid-hill areas, hybrid maize varieties produced 20%–30% more grain than OPVs. Higher profitability resulted from the usage of hybrids, particularly for commercial maize producers. In 2021, the Nepal Agricultural Research Council (NARC) also found that hybrid tomato cultivars like Srijana and Manisha performed better than conventional cultivars in terms of production, fruit size, and disease resistance, which helped to increase farmer income in periurban regions [65]. In addition to higher uniformity, several hybrid cultivars have better nutritional characteristics. Research has demonstrated that certain hybrid tomato and sweet pepper varieties can have larger yields and improved fruit quality than traditional and organic varieties, especially under drought stress circumstances [66].

## 11. Negative Impact of Hybrid Varieties in Crop Production

Plants that are genetically identical in hybrids lack variation, making them more susceptible to new diseases and insects. Sometimes, low genetic variation can cause genetic mutations. The F1, grown from hybrid seeds, is either sterile or seedless. Therefore, the farmer must purchase seed annually, thereby increasing cost of crop production [67]. Hybrids have a significant impact on crop genetic diversity. The increasing use of high-productive hybrid varieties can lead to the displacement of traditional landraces. The reduction in crop genetic diversity due to hybrid varieties can make crops more vulnerable to insects and pests, leading to significant yield loss and decreased resilience to climate change [68]. Soil degradation and soil pollution are other major issues created by the heavy use of inorganic fertilizer and pesticides in hybrid cropping systems [59]. Some cases of crop failure and reduced yield with hybrid varieties particularly in maize was reported by [69]. The extensive adoption of hybrid rice varieties is causing indigenous rice varieties like Jethobudo, Anadi, and Mansuli to perish in Nepal's Terai and mid-hill areas. Despite having a high yield, farmers said that hybrid rice had poorer grain quality and cooking qualities, which decreased customer demand and market pricing [70]. OPVs were less susceptible to downy mildew and Turcicum leaf blight than hybrid maize types CP-666 and Pioneer-30V92, which are widely grown in the mid-hill regions. Farmers who had spent money on pricey hybrid seeds and agrochemicals experienced financial hardship as a result of crop failures brought on by frequent disease outbreaks [71]. Commercial hybrid vegetable cultivation in Dhading and Chitwan has resulted in an overabundance of synthetic pesticides and fertilizers, which has decreased soil fertility and contaminated water supplies. Although hybrid crops produced good yields at first, farmers said that prolonged cultivation decreased soil productivity, requiring them to use more chemicals and increasing production expenses [72, 73]. Bacterial wilt outbreaks in Srijana hybrid tomato crops caused large losses for farmers in Kaski. Even though Srijana did well in controlled environments, field-level cultivation revealed lower production and increased susceptibility to illnesses, which made many farmers switch back to native tomato cultivars that needed less agrochemicals [74]. These results demonstrate that hybrid varieties provide serious difficulties for Nepali farmers, especially those with limited resources. The frequency of these detrimental effects emphasizes the necessity of a well-rounded strategy that combines hybrid technology with the preservation of native cultivars to guarantee resilient and sustainable farming systems.

## 12. Response of Hybrids With Disease, Insects, and Pests

It has been suggested that rice hybrids are inherently more susceptible than conventional varieties to insect pests and diseases [75]. Many studies suggest that hybrid rice has been highly susceptible to damage from a range of insects [76]. Rice hybrid varieties are more vulnerable to disease epidemics and insect outbreaks. Many reports from China showed that rice hybrid varieties are highly susceptible to or vulnerable to the following insects: yellow stem borer (*Scirpophaga incertulas*), striped stem borer (*Chilo suppressalis*), rice leaf folder (*Cnaphalocrocis medinalis* Guenee), and rice skipper (*Pelopidas mathias*) [77]. With the incidence of insect pests, especially stem borers, and diseases more frequently appearing in hybrid rice than in inbred varieties, the major diseases that occur in hybrid rice in China are rice blast (*Magnaporthe oryzae*), sheath blight (*Rhizoctonia solani*), and bacterial blight (*Xanthomonas oryzae* pv. Oryzae) [78]. To control pest and disease outbreaks in hybrid rice agriculture, Nepal uses a number of mitigating techniques. To lessen dependency on chemical pesticides, Integrated Pest Management (IPM) strategies are encouraged, such as crop rotation, biological control with natural predators (e.g., *Trichogramma* wasps for stem borers), and the use of biopesticides [79]. Additionally, breeding initiatives and partnerships with foreign research institutes are being used to create resistant hybrid rice cultivars. To fight bacterial blight and sheath blight, systemic fungicides and microbial bioagents such as *Pseudomonas fluorescens* have been promoted [80]. To reduce the burden of pests and diseases, farmers are also implementing cultural techniques including balanced fertilizer application, good field hygiene, and efficient irrigation management. To guarantee sustainable hybrid rice production, information on best practices is regularly shared by the Nepal Agricultural Research Council (NARC) and agricultural extension services.

## 13. Challenges and Constraints

The hybrid seeds used by farmers for both grain and vegetables are mostly imported from outside the country. The risks of relying on imported seeds extend beyond the country's food and economic security [31]. However, the use of hybrid seeds helps to increase productivity, but the cost of production is higher as compared to indigenous seeds. Hybrid seed procurement is not reliable because farmers never receive the seed they ordered. This is due to Nepal's weak seed distribution system, in which farmers have no alternative but to buy from agrovets [3]. There are numerous problems with cultivating hybrid cultivars, including high soil nutrient exhaustion, high input requirements, poor milling attributes, and poor storability [81].

The main challenges Nepal faces in hybrid cultivation, particularly in maize, include a shortage of technical manpower, unscientific farming practices, a lack of expertise, an inadequate package of practices, limited investment from both the public and private sectors, the unavailability and high cost of inputs, and insufficient research on hybrid crops [37]. Small-scale farmers encounter numerous problems when it comes to hybrid varieties, such as insufficient initial investment, the absence of subsidies, limited access to credit, and the requirement for high inputs [11]. A significant proportion of agrovet proprietors (2200 registered in NSB) are from nonagriculture backgrounds and do not possess sufficient technical expertise to effectively handle and deliver accurate information to farmers regarding hybrid seeds. NSP is somehow progressive, but it requires some amendments like licensing the private seed laboratories, as reported by Thapa [82].

Nepal has a limited number of hybrid varieties available. Every year, Nepal imports around 4000 metric tons of hybrid seeds for rice, maize, and vegetables, at a total cost of over 15 million USD. Because there were few plant breeders participating in agricultural research, farmers had to pay a high price for hybrid varieties imported from abroad [83]. For growers, poor investment in demand-driven breeding and a lack of competition among seed companies are major obstacles to improved varieties. Poor involvement of institutes like NAARC, AFU, and TU in agricultural research and the absence of improved labs and trained scientists in research and breeding due to poor funding from the government in research are additional important challenges making hybrid variety development challenging [25]. Nepal currently does not have effective regulations in place to support genetic innovation, encompassing areas such as genetics and breeding, utilizing genetic diversity, and utilizing novel biotechnological features like golden rice and drought-tolerant wheat. These advancements hold significant potential for Nepal's future development. Nepal may take lessons from our neighboring nations, like India, China, the Philippines, and Bangladesh, who are highly engaged in new technologies of crop genetics, hybrid breeding, adequate plant variety protection (PVP) regulations, and private-sector entrepreneurship [84]. Ongoing efforts must concentrate on bolstering the seed distribution network, funding regional hybrid seed production, and offering farmers and agrovet owners technical training in order to overcome these obstacles. Growing public–private collaborations can encourage innovation and lessen reliance on imported seeds. Furthermore, improving the adoption and sustainability of hybrid seeds in Nepal will require increasing funding for agricultural research, encouraging the participation of agricultural institutions in breeding programs, and developing efficient laws for genetic innovation and PVP. Nepal might benefit from studying neighboring nations like Bangladesh, China, India, and the Philippines, which have successfully established strong regulatory frameworks, hybrid breeding programs, and cutting-edge crop genetics technology [85]. Nepal might also look into working with foreign groups to encourage sustainable farming methods, provide access to contemporary technology, and promote the development of genetic research capability. In order to empower smallholder farmers and advance long-term agricultural sustainability, the government should think about providing financial incentives or subsidies for the development of hybrid seeds locally and the use of sustainable farming methods.

## 14. Future Prospects

Nepal has significant potential to expand its hybrid seed production and marketing to meet the growing domestic demand and reduce expensive imports. Here are some key future prospects for hybrid seeds and varieties in Nepal.

### 14.1. Increasing Private-Sector Engagement

The Nepalese government has opened up seed variety development for private seed companies, allowing them to conduct varietal research, release new varieties, and market them under their own brands. This policy will expand local production and businesses on hybrid and nonhybrid seeds, boost productivity, foster a competitive seed sector, attract private investment, and create more jobs in the emerging seed business. The policy interventions provide opportunities for local seed companies to capitalize on hybrid seed opportunities [30]. One example is Lumbini Seed Company, which has expanded hybrid maize seed production from 1 ha to 25 ha within 3 years, with a goal of producing and selling 200 metric tons of hybrid maize by 2025 [86].

### 14.2. Reducing Reliance on Imports

Nepal currently imports a significant amount of hybrid seeds annually from India, China, and abroad to suit the expanding domestic demand, costing billions of Rupees [25]. Developing suitable hybrid varieties and making their seeds available when and where needed is crucial to reducing imports. The National Seed Vision (2013–2025) aims to encourage the private sector to develop and promote hybrid seeds as import substitutions and build self-reliance [28]. The potential maize seed requirement for Nepal is around 20,000 metric tons, which creates an estimated $80 million market if the SRR for maize is to be 100% [30].

### 14.3. Increasing Crop Production and Productivity

Hybrid varieties are a cost-effective strategy for increasing the production and productivity of major crops in Nepal, such as cereals and vegetables. These hybrids offer advantages like high productivity, uniformity, better transport quality, and resistance to abiotic and biotic stresses. About 73% of vegetables are covered by hybrid varieties, and their use is increasing even in small pockets [33]. Currently, only 10%–15% of Nepal's maize-growing area is covered by hybrid seeds, leaving the balance for OPVs. Increasing hybrid seed adoption can significantly boost on-farm productivity, which is currently around 2.8 metric tons per hectare for maize [86].

### 14.4. Strengthening Seed Systems and Enabling Policies

Nepal needs to enhance its seed systems to maximize the potential of hybrid seeds. This includes building stakeholder capacity, strengthening agricultural input markets and extension systems, improving investment incentives, and developing a well-designed product life cycle management process to improve SRRs [31]. The National Seed Vision (2013–2025) provides a pragmatic and holistic long-term vision for the development of Nepal's seed sector, identifying gaps, opportunities, and strategies for variety development, seed multiplication, processing, marketing, quality control, and use [28]. Breeding programs, farmer education, and the creation of climate-resilient hybrid varieties should be the main priorities of agricultural universities and research institutes like Tribhuvan University and AFU. High-quality hybrid seeds must be made available, and farmers must get technical assistance from private seed businesses and agrovets. The entire seed system may be strengthened by the crucial roles that farmers' organizations and cooperatives can play in information exchange, seed distribution, and seed multiplication. Nepal's advancement in seed innovation and food security can potentially be accelerated by partnerships with nearby nations that have sophisticated hybrid breeding programs and international research groups.

## 15. Conclusion

In Nepal, hybrid seeds are currently becoming popular. Hybrid varieties are developed by crossing between two distinct, dissimilar parents. Hybrid seeds have greater yield potential than nonhybrid seeds. Hybrid seeds have both positive and negative aspects. The positive aspects are high-yield advantages, which is a strong factor that encourages farmers to adopt hybrid seed, and uniformity among different agronomic traits. The negative aspects of hybrid seed are that farmers pay a high amount for purchasing hybrid seed, managing fertilizer and pesticides, suppressing local agrobiodiversity, and the chance of a high incidence of insects, diseases, and pests. Each year, Nepal invests a huge amount of money in importing hybrid seed for cereal and vegetables from outside the country. The Nepalese government should place emphasis on research and development of hybrid varieties while also collaborating with the private sector. Nepal can also learn from neighboring countries (India and China) in terms of the technology they have adopted for seed sector development. Stakeholders and policymakers are concerned about formulating essential policies, simplifying existing seed policies, and breeding rules and regulations. They can use these findings to conduct further research and develop hybrid seeds.

## Figures and Tables

**Figure 4:**
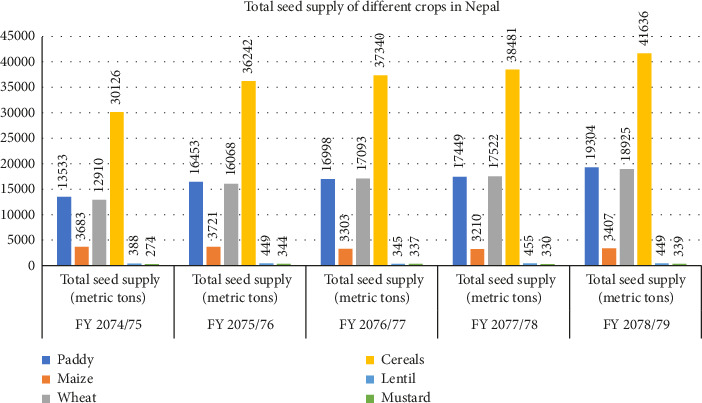
Total seed supply of different crops.

**Table 1:** The current status and projected area under hybrid seeds.

	Year (A.D.)	Maize area (ha)	Maize seed reqd. (mt)	Rice area (ha)	Rice seed reqd. (mt)	Vegetable area (ha)	Vegetable seed reqd. (mt)
Status	2010	85,000	1275	32,000	640	30,000	30

Projection	2015	120,000	1800	50,000	1000	40,000	40
	2020	180,000	2700	100,000	2000	70,000	70
	2025	250,000	3750	150,000	3000	90,000	90

Source: [28].

## Data Availability

Data will be made available upon request.
